# Emerging Serological Biomarkers for Autoimmune Hepatitis

**DOI:** 10.3390/jcm15135123

**Published:** 2026-07-01

**Authors:** Kazumichi Abe, Jun Wada, Hiromasa Ohira

**Affiliations:** Department of Gastroenterology, Fukushima Medical University School of Medicine, Fukushima City 960-1295, Japan

**Keywords:** autoimmune hepatitis, human protein microarray, autoantibody, docking protein 2, mitochondrial ribosomal protein S27

## Abstract

Autoimmune hepatitis (AIH) lacks a disease-specific diagnostic biomarker, particularly for patients who present with acute onset, normal IgG levels, or seronegative profiles. Recent advances in high-throughput autoantibody profiling technologies have enabled the systematic discovery of novel AIH-associated autoantigens. This review summarizes emerging autoantibodies that have been identified through proteome-wide screening approaches, including human protein microarrays and phage immunoprecipitation sequencing. Particular attention is given to anti-docking protein 2 (DOK2) and anti-mitochondrial ribosomal protein S27 (MRPS27) antibodies, which have shown promising diagnostic performance. Importantly, the combined assessment of these autoantibodies markedly improves the diagnostic sensitivity for AIH and may be useful for screening or clinical triage, although its reduced specificity limits its use as a stand-alone confirmatory test. We also discuss how the integration of serological biomarkers with molecular pathology and spatial immune analysis may advance the understanding of AIH pathogenesis. These developments highlight the potential of proteome-wide autoantibody discovery to refine diagnostic strategies for AIH and provide new insights into disease mechanisms.

## 1. Introduction

Autoimmune hepatitis (AIH) is a chronic immune-mediated liver disease that is characterized by progressive hepatic inflammation and the presence of circulating autoantibodies. Although AIH is considered a rare disease, its recognition has increased worldwide, and its clinical presentation is highly heterogeneous [1]. AIH can affect both children and adults, shows a female predominance, and may present as asymptomatic chronic hepatitis, acute hepatitis-like onset, acute severe hepatitis, or established cirrhosis. Because delayed diagnosis may result in progressive fibrosis, cirrhosis, or liver failure, reliable biomarkers that facilitate earlier recognition and diagnostic stratification are clinically needed [2,3,4,5].

Despite substantial advances in understanding the immunopathology of AIH, a single disease-specific diagnostic marker is still lacking. Current diagnostic strategies that are recommended by the international guidelines from the European Association for the Study of the Liver (EASL) and the American Association for the Study of Liver Diseases (AASLD) rely on an integrated evaluation of serum autoantibodies, serum immunoglobulin G (IgG) levels, histological findings, and the exclusion of other liver diseases, rather than on a single definitive diagnostic test [2,3,4,5]. In addition, the simplified diagnostic criteria proposed by the International Autoimmune Hepatitis Group have improved the practical classification of patients with probable or definite AIH in clinical settings [6]. The simplified criteria are primarily classification-oriented rather than early screening tools because they require histological assessment and depend partly on conventional autoantibody positivity and serum IgG elevation.

However, the use of conventional autoantibodies has several limitations. AIH is traditionally classified into type 1 and type 2 according to autoantibody profiles. Type 1 AIH is characterized mainly by antinuclear antibodies (ANAs) and/or anti-smooth muscle antibodies (ASMAs) positivity and is the predominant form in adults, whereas type 2 AIH is associated with anti-liver–kidney microsome type 1 (LKM-1) and/or anti-liver cytosol type 1 (LC-1) antibodies and is more frequently observed in children and young patients [7]. ANAs and ASMAs, which are commonly detected in patients with type 1 AIH, exhibit only moderate diagnostic accuracy. Furthermore, approximately 10% of patients with AIH have normal serum IgG levels, and some patients lack detectable conventional autoantibodies, a condition referred to as seronegative AIH [8,9,10,11]. Such atypical presentations may delay the diagnosis and complicate clinical decision-making.

Recent technological advances in high-throughput serological profiling, including human protein microarrays, peptide/protein microarrays, phage immunoprecipitation sequencing (PhIP-Seq), and synthetic human peptidome or virome-based profiling, have enabled the comprehensive exploration of disease-associated autoantibodies [12,13,14,15]. These proteome-wide approaches provide new opportunities to identify previously unrecognized autoantibody targets and may contribute to improving the diagnostic accuracy for AIH and the understanding of disease mechanisms.

The diagnostic difficulty of AIH is particularly evident in patients who present with acute hepatitis-like onset, normal serum IgG levels, or negative conventional autoantibodies. In such cases, the clinical and biochemical features may overlap with those of drug-induced liver injury, viral hepatitis, metabolic dysfunction-associated steatotic liver disease (MASLD), or cholestatic autoimmune liver diseases. Given that early recognition of AIH is important for the timely initiation of immunosuppressive therapy, delayed diagnosis may lead to prolonged hepatic inflammation and progression to severe liver injury. Therefore, biomarkers that can complement existing diagnostic criteria are clinically valuable, especially for patients who do not present with typical serological patterns. Emerging autoantibodies should not be regarded as substitutes for histological assessment but rather as adjunctive tools that may increase diagnostic confidence in patients with clinically suspected but serologically atypical AIH. Liver biopsy remains essential for the diagnosis of AIH because it allows direct assessment of interface hepatitis, lymphoplasmacytic infiltration, hepatocellular injury patterns, fibrosis stage, and histological features suggesting alternative or overlapping liver diseases [3,4,5]. However, histological assessment has several limitations, including its invasive nature, sampling variability, interobserver differences, and limited suitability for repeated monitoring [4,5]. In contrast, circulating autoantibodies are minimally invasive, repeatable, and clinically accessible biomarkers that can support early diagnostic evaluation. Although autoantibody-based biomarkers cannot replace liver biopsy, they remain attractive adjunctive tools, particularly for patients with seronegative AIH, normal serum IgG levels, acute-onset disease, or diagnostically ambiguous presentations [6,8,9,10,11].

In this review, we summarize recent advances in emerging serological biomarkers for AIH, with a particular emphasis on autoantibodies that have been identified through proteome-wide screening technologies. We also discuss their potential diagnostic implications and future perspectives for biomarker-based diagnosis.

## 2. Diagnostic Framework and Scoring Systems

AIH cannot be diagnosed by a single disease-specific test. Instead, its diagnosis relies on an integrated assessment of clinical presentation, biochemical abnormalities, immunological features, and histological findings [3,4]. Elevated levels of serum aminotransferases and hypergammaglobulinemia represent fundamental biochemical characteristics, whereas immunological hallmarks include ANA, ASMA, and anti-LKM-1 positivity.

To standardize the diagnosis, the International Autoimmune Hepatitis Group (IAIHG) proposed a revised diagnostic scoring system [16,17] and a simplified scoring system [6]. The revised scoring system allows detailed evaluation by incorporating autoantibody data, IgG levels, histological findings, the exclusion of viral hepatitis, drug exposure, and alcohol intake but is relatively complex for routine practice [16]. In contrast, the simplified scoring system consists of four components—autoantibody data, IgG levels, histological findings, and the exclusion of viral hepatitis—and is widely used as a practical tool in daily clinical settings to classify patients as having probable or definite AIH [6]. Recently, the 2025 EASL Clinical Practice Guidelines proposed an updated version of simplified diagnostic criteria to better reflect current laboratory practices, particularly the widespread use of HEp-2 cells and ELISA-based autoantibody assays [4]. In the updated system, autoantibody scoring has been refined to incorporate different testing platforms, and strongly positive titres, as assessed with HEp-2 cells or ELISAs, are specifically acknowledged. In addition, substitution of conventional histological definitions with the 2022 International AIH Pathology Group (IAHPG) criteria has been suggested to improve diagnostic sensitivity, particularly in cases with acute or atypical presentations. Although external validation is still limited, these refinements may enhance real-world diagnostic performance.

Despite these advances, the diagnostic scoring systems have inherent limitations. The revised IAIHG score is comprehensive and useful in research settings or in diagnostically complex cases, but its complexity may limit its routine use in daily clinical practice. The simplified score is easier to apply and has become widely accepted; however, its performance depends substantially on the presence of conventional autoantibodies and elevated IgG levels. As a result, patients with seronegative AIH or AIH with normal serum IgG levels may fail to obtain sufficient serological points despite having compatible clinical and histological features. This limitation is particularly relevant in acute-onset AIH, in which typical features of chronic AIH, such as marked hypergammaglobulinemia or established interface hepatitis, may be absent or less prominent at the time of presentation. Therefore, diagnostic criteria should be interpreted in the context of the overall clinical probability of AIH rather than used as rigid exclusion tools. Conversely, overly loose application of diagnostic criteria may reduce specificity and lead to misdiagnosis, particularly in patients with drug-induced liver injury, viral hepatitis, MASLD, or cholestatic autoimmune liver diseases. This is clinically important because unnecessary immunosuppressive therapy can expose patients to substantial risks. Therefore, scoring systems should support, but not replace, careful clinical judgment.

## 3. Conventional Autoantibodies and Their Diagnostic Limitations for AIH

Autoantibodies have long been recognized as a central component of the diagnostic framework for AIH. Among them, ANAs and ASMAs are most frequently detected in patients with type 1 AIH and are therefore widely used as diagnostic markers in clinical practice. However, these antibodies are not disease-specific and may also be detected in patients with other autoimmune or inflammatory conditions [18].

Several additional autoantibodies have been reported in patients with AIH, including anti-soluble liver antigen/liver–pancreas (SLA/LP) antibodies, anti-LKM-1 antibodies, and anti-LC-1 antibodies [19,20]. Among these, anti-SLA/LP antibodies are considered highly specific for type 1 AIH and are associated with severe disease and an increased risk of relapse. In contrast, anti-LKM-1 and anti-LC-1 antibodies are associated mainly with type 2 AIH. These antibodies are widely used in clinical practice and are incorporated into diagnostic scoring systems for AIH (Table 1). A meta-analysis reported that ANA has moderate diagnostic sensitivity and specificity, ASMA has moderate sensitivity with high specificity, and anti-SLA/LP antibodies have low sensitivity but very high specificity for AIH [18].

The clinical interpretation of conventional autoantibodies requires caution. ANAs and ASMAs are highly useful first-line serological markers for type 1 AIH, but they are not specific to AIH. Low-titre ANA positivity may be observed in patients with other autoimmune diseases, chronic viral hepatitis, metabolic liver disease, and cholestatic liver diseases and even in healthy individuals. Similarly, ASMA positivity may vary depending on the assay method, cut-off value, and laboratory expertise. Therefore, ANA or ASMA positivity alone is insufficient to establish a diagnosis of AIH without compatible biochemical, histological, or clinical findings. In contrast, anti-SLA/LP antibodies are highly specific for type 1 AIH and may support AIH diagnosis when present, but their relatively low prevalence limits their sensitivity as screening markers. Anti-LKM-1 and anti-LC-1 antibodies are important for the diagnosis of type 2 AIH, particularly in children and young patients, whereas type 2 AIH is uncommon in adult Japanese cohorts. These characteristics indicate that conventional autoantibodies remain indispensable but are insufficient to resolve all diagnostically ambiguous cases.

Nevertheless, the prevalence of these autoantibodies varies considerably among populations. In Japanese patients with AIH, anti-SLA/LP antibodies are detected in approximately 15–20% of cases, anti-LKM-1 antibodies in approximately 10% of cases, and anti-LC-1 antibodies in less than 5% of cases. Consequently, reliance on conventional autoantibodies alone is insufficient for accurate diagnosis in a subset of patients, particularly those with atypical clinical presentations.

These diagnostic challenges highlight the need for additional biomarkers that can complement existing serological markers and improve diagnostic confidence in patients with suspected AIH.

## 4. Emerging Autoantibodies Identified by Targeted and Unbiased Approaches

Emerging autoantibodies for the diagnosis of AIH can be broadly classified according to their discovery strategy. Some have been identified through targeted approaches based on candidate antigens that are related to immune regulation, hepatocellular injury, or intracellular metabolism. Others have been discovered through unbiased high-throughput platforms that screen large numbers of human proteins or peptides without prior assumptions regarding antigen specificity. This distinction is important because candidate-based approaches are biologically hypothesis-driven, whereas unbiased approaches may reveal unexpected autoantigens and provide new insights into disease mechanisms.

Unbiased proteome-wide technologies, such as phage immunoprecipitation sequencing and large-scale protein or peptide microarrays, have emerged as powerful platforms for the systematic discovery of disease-associated autoantibodies, enabling the comprehensive characterization of humoral immune repertoires beyond conventional candidate-based approaches [12,13]. Several novel autoantibodies, such as anti-programmed cell death-1 (PD-1), anti-phosphoenolpyruvate carboxykinase 2 (PCK2), antinucleosome, and anti-phosphoglycerate dehydrogenase (PHGDH) antibodies (Table 1), have been reported to have potential diagnostic utility for AIH [21,22,23]. These autoantibodies have been individually validated via standardized immunoassays, such as enzyme-linked immunosorbent assays (ELISAs), based on predefined candidate antigens. Although these autoantibodies have not been established as disease-specific diagnostic markers, they have attracted attention because they may reflect specific aspects of AIH pathophysiology, including hepatocellular injury, dysregulation of immune homeostasis, or disruption of intracellular metabolic pathways.

Anti-PD-1 antibodies have been reported to function as autoantibodies targeting immune checkpoint molecules and may reflect the breakdown of immune homeostasis, which is related to impaired regulation of T-cell activation. In contrast, anti-PCK2 and anti-PHGDH antibodies target intracellular metabolic enzymes and may represent secondary autoimmune responses that are induced by exposure to antigens associated with hepatocellular injury or metabolic stress. Antinucleosome antibodies recognize DNA–histone complexes that are exposed during apoptosis or cell death and are correlated with disease activity and amplification of immune responses in patients with AIH. Collectively, these autoantibodies are likely induced during hepatocellular damage and excessive immune activation, which highlights the multilayered nature of immune dysregulation during AIH. Although most of these antibodies have not yet been incorporated into diagnostic criteria, further investigation is warranted to clarify their roles as complementary markers for assessing disease activity and understanding pathogenic mechanisms.

In parallel, autoantibodies that have been identified through unbiased screening approaches—such as human protein microarrays and PhIP-Seq—have been increasingly reported. Klepper and colleagues performed comprehensive autoantibody profiling by using PhIP-Seq in 115 patients with AIH and identified novel autoantibody targets [24]. In addition to the established type 1 AIH-specific anti-SLA/LP antibodies, antibodies against relaxin family peptide receptor 1 (RXFP1) and cross-reactive antibodies with the human herpesvirus 6 (HHV-6) U27 protein were detected. Both anti-RXFP1 antibodies and anti-HHV-6 U27 cross-reactive antibodies were observed in approximately 6–8% of patients with AIH but were rarely detected in healthy controls or patients with other liver diseases. Notably, these autoantibodies were also detected in a subset of seronegative AIH patients.

Furthermore, Taubert and colleagues employed a human protein microarray-based approach to identify polyreactive IgG (pIgG) antibodies that bound to human huntingtin-interacting protein 1-related protein/bovine serum albumin (HIP1R/BSA) [25]. Unlike conventional autoantibodies that are directed against specific antigens, pIgG represents a novel immunological concept reflecting exaggerated immune activation in patients with AIH. Analysis of 1568 patients with AIH demonstrated that compared with conventional autoantibodies, pIgG had higher diagnostic specificity (up to +25%) and sensitivity (up to +20%) and was positive in 88% of seronegative AIH patients and 71% of patients with normal IgG levels. These findings suggest that pIgG may serve as a promising adjunctive biomarker for the diagnosis of seronegative AIH. However, because Japanese patients with AIH were not included in these cohorts, a comprehensive exploration of disease-specific autoantibodies in these patients remains necessary.

## 5. Proteome-Based Discovery of Novel Autoantibodies

Comprehensive proteome-wide screening technologies have recently enabled the systematic exploration of disease-associated autoantibodies in patients with autoimmune diseases, including AIH. Among these approaches, human protein microarray platforms allow large-scale interrogation of antibody reactivity against thousands of human proteins and have become powerful tools for identifying previously unrecognized autoantigens.

**Figure 1:**
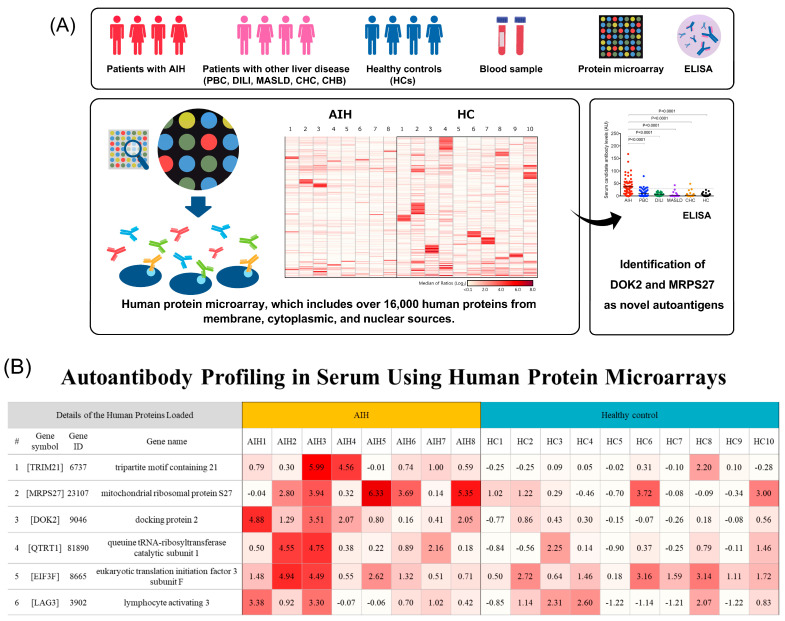
Identification of anti-DOK2 and anti-MRPS27 autoantibodies in patients with autoimmune hepatitis via a protein microarray: (**A**) Schematic workflow of the protein microarray assay. Serum samples from patients with AIH or other liver diseases and from healthy controls were screened for autoantibodies by using a human protein microarray containing more than 16,000 human proteins. Candidate autoantigens identified in the screening step were subsequently validated by ELISAs. (**B**) Representative heatmap of protein microarray signals comparing patients with autoimmune hepatitis (AIH) and healthy controls (HCs). Each row represents an individual protein, and each column represents an individual serum sample. DOK2 and MRPS27 were identified as candidate autoantigens that were significantly more reactive in serum from patients with AIH than in that from healthy controls [27].

To facilitate the identification of AIH-specific autoantibodies, comprehensive autoantibody profiling was conducted by using a human protein microarray platform developed under the Fukushima Pharmaceutical-Related Industry Support Project. This platform comprises more than 16,000 human cDNAs encoding membrane, cytoplasmic, and nuclear proteins, which were synthesized by using a wheat germ cell-free expression system, thereby enabling unbiased detection of previously unrecognized autoantibody targets (Figure 1A). The advantage of this platform is that it enables the simultaneous assessment of antibody reactivity against a broad range of human proteins, including intracellular proteins that would not necessarily be selected in conventional candidate-based studies. The use of a wheat germ cell-free expression system may also facilitate the production of diverse human proteins while avoiding some of the limitations associated with bacterial expression systems. However, as with other protein microarray approaches, candidate signals require careful validation because the antigen conformation, posttranslational modifications, and nonspecific binding may influence the screening results. Therefore, independent validation by an ELISA and comparison with disease control groups are essential steps for determining whether identified autoantibodies have disease relevance.

By using this human protein microarray system, serum autoantibody profiles of patients with AIH and healthy controls were systematically analysed. Comparative profiling revealed multiple autoantibody candidates that were significantly enriched in the AIH group. Among these, docking protein 2 (DOK2) [26] and mitochondrial ribosomal protein S27 (MRPS27) [27] emerged as the leading candidate antigens on the basis of their disease specificity, signal intensity, and immunological plausibility (Figure 1B).

## 6. Novel Autoantibodies Identified by Proteome-Wide Screening

### 6.1. Anti-DOK2 Antibodies

Subsequent validation was performed in an independent cohort of patients with AIH together with several disease control groups—including primary biliary cholangitis (PBC), drug-induced liver injury (DILI), metabolic dysfunction-associated steatotic liver disease (MASLD), and viral hepatitis—as well as healthy controls. Serum anti-DOK2 antibody levels were measured by an ELISA with a recombinant DOK2 protein, with cut-off values determined on the basis of their distribution in healthy controls. The anti-DOK2 antibody levels were significantly higher in patients with AIH than in those with other liver diseases and in healthy individuals, demonstrating good diagnostic performance, with AUROC values ranging from approximately 0.87–0.90. Notably, anti-DOK2 antibodies were detected in approximately 65–80% of AIH patients, including those with normal serum IgG levels and ANA-seronegative AIH, suggesting their utility in diagnostically challenging cases.

DOK2 is an adaptor protein that is expressed mainly in haematopoietic cells and is involved in the regulation of immune receptor signalling. Because DOK2 is involved in signalling pathways related to macrophage activation, T-cell responses, and innate immune regulation, the identification of anti-DOK2 antibodies in patients with AIH may have biological relevance beyond diagnostic utility. In particular, DOK2 may function as a regulatory molecule that modulates inflammatory signalling rather than as a hepatocyte-specific structural antigen. This feature distinguishes anti-DOK2 antibodies from autoantibodies directed against intracellular hepatocellular components and suggests that anti-DOK2 reactivity may reflect immune dysregulation within the intrahepatic inflammatory environment. The associations between anti-DOK2 antibody levels, serum IgG concentrations, and histological inflammatory activity support this interpretation. Thus, anti-DOK2 antibodies may serve not only as adjunctive diagnostic markers but also as serological indicators of immune activation in patients with AIH. These observations indicate an association between anti-DOK2 antibodies and immune activation, but they do not establish a direct pathogenic role for these antibodies.

In this study, the clinical significance of anti-DOK2 antibodies was evaluated through an integrated analysis of serological parameters, histopathological findings, and immune cell levels. Serum anti-DOK2 antibody levels were significantly positively correlated with serum IgG concentrations, indicating that higher antibody titres are associated with cases of more immunologically active AIH. In addition, anti-DOK2 antibody levels were positively correlated with histological inflammatory activity, as assessed by liver biopsy, suggesting that these autoantibodies reflect the degree of intrahepatic immune-mediated inflammation at the serological level. Multivariate analyses further demonstrated that serum IgG levels were independently associated with elevated anti-DOK2 antibody titres, supporting the notion that anti-DOK2 antibodies are not merely nonspecific byproducts of immune activation but are closely linked to the immunopathogenesis of AIH [26].

### 6.2. Anti-MRPS27 Antibodies

More recently, anti-MRPS27 antibodies have been identified as another novel serological biomarker for AIH. These antibodies are detected in approximately 60% of patients and show moderate sensitivity with relatively high specificity. MRPS27 is a component of the mitochondrial ribosomal small subunit that is involved in mitochondrial protein synthesis [27].

MRPS27 and DOK2 represent distinct types of autoantigens. As a mitochondrial ribosomal protein, MRPS27 may be linked to mitochondrial stress responses in injured hepatocytes. In patients with AIH, immune-mediated hepatocellular injury may promote mitochondrial dysfunction, the release or exposure of intracellular antigens, and subsequent autoantibody production. Immunohistochemical observations showed increased cytoplasmic MRPS27 expression in injured hepatocytes, thus supporting the concept that MRPS27-related autoimmunity may be associated with hepatocellular damage. However, serum anti-MRPS27 antibody titres do not appear to directly reflect the severity of individual histological inflammatory features, suggesting that these antibodies may represent a broader injury-associated immune response rather than a single microscopic lesion. Similarly, anti-MRPS27 antibodies may reflect hepatocellular injury-associated antigen exposure rather than direct pathogenic autoimmunity. This distinction may explain why anti-MRPS27 antibodies complement anti-DOK2 antibodies in the serological diagnosis of AIH.

### 6.3. Complementary Diagnostic Value of Anti-DOK2 and Anti-MRPS27 Antibodies

Importantly, the combined assessment of anti-DOK2 and anti-MRPS27 antibodies markedly improves the diagnostic sensitivity for AIH, although this increase in sensitivity is accompanied by a reduction in specificity. These findings suggest that the two antibodies may play complementary roles as adjunctive serological markers, particularly for screening or clinical triage in patients with suspected AIH. The complementarity of anti-DOK2 and anti-MRPS27 antibodies may be explained by their different biological backgrounds. Anti-DOK2 antibodies may be more closely associated with immune cell-mediated inflammation and systemic immunological activity, whereas anti-MRPS27 antibodies may reflect mitochondrial antigen exposure in injured hepatocytes. Therefore, the two antibodies may capture partially distinct immunopathological aspects of AIH. This conceptual distinction provides a rationale for combined testing and supports the use of these antibodies as a biomarker panel rather than as isolated single markers.

## 7. Diagnostic Implications of a Combined Autoantibody Assessment

To further evaluate the diagnostic performance of these autoantibodies, we summarized the ELISA-based validation data for anti-DOK2 and anti-MRPS27 antibodies. The diagnostic performance of anti-DOK2 and anti-MRPS27 antibodies as individual markers has been reported in the original studies [26,27]. As shown in Table 2, anti-DOK2 antibodies alone demonstrated a sensitivity of 64.2% (95% CI, 52.2–74.6) and a specificity of 91.3% (95% CI, 84.2–95.3), with a positive predictive value (PPV) of 82.7% and a negative predictive value (NPV) of 79.7%. Anti-MRPS27 antibodies showed a comparable sensitivity of 61.2% (95% CI, 49.2–72.0) but a slightly lower specificity of 84.5% (95% CI, 76.3–90.2). In the present review, we additionally evaluated the potential diagnostic implications of a combined “DOK2 or MRPS27” strategy using an either-positive criterion. With this approach, the diagnostic sensitivity increased to 92.5% (95% CI, 83.7–96.8), with a high NPV of 94.2%, although the specificity decreased to 78.6% (95% CI, 69.8–85.5). These findings suggest that the combined assessment of anti-DOK2 and anti-MRPS27 antibodies may provide a highly sensitive screening or triage strategy for AIH, whereas anti-DOK2 antibodies alone may serve as a more specific adjunctive marker.

The interpretation of combined autoantibody testing should be based on the intended clinical use. A positive marker with high specificity is useful for increasing diagnostic confidence, whereas a negative marker or combination with high sensitivity and a high NPV is useful for reducing the likelihood of disease. From this perspective, anti-DOK2 antibodies may be suitable as relatively specific adjunctive markers, whereas the combined DOK2-or-MRPS27 strategy may be more appropriate as a sensitive screening approach in patients with suspected AIH. Nevertheless, the decrease in specificity that was observed with either positive criterion indicates that positive results should not be interpreted in isolation. Rather, data for these antibodies should be integrated with those on clinical presentation, liver biochemistry, serum IgG levels, conventional autoantibodies, and histological findings. Because the “either positive” strategy increases sensitivity at the expense of specificity, false-positive results may occur in patients with inflammatory liver diseases, autoimmune overlap syndromes, immune checkpoint inhibitor-related hepatitis, chronic viral hepatitis, and severe MASLD/NASH. Therefore, positive anti-DOK2 or anti-MRPS27 results should not be interpreted as diagnostic by themselves but should be integrated with clinical context, conventional autoantibodies, IgG levels, exclusion of competing aetiologies, and histology.

**Figure 2:**
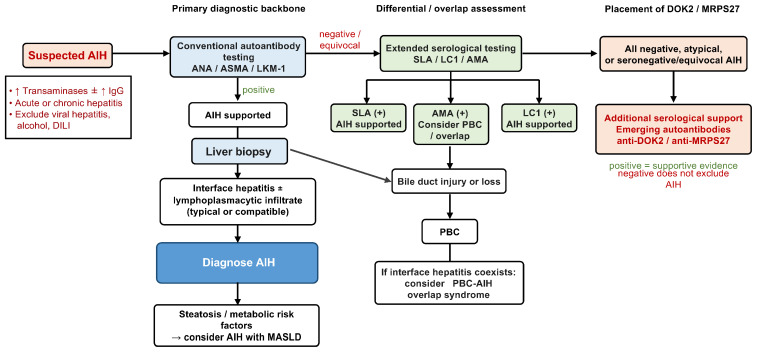
Proposed diagnostic algorithm for autoimmune hepatitis incorporating additional serological support from emerging autoantibodies. Patients with suspected autoimmune hepatitis (AIH), based on elevated transaminases with or without increased IgG after exclusion of viral hepatitis, alcohol-related liver injury, and drug-induced liver injury, should undergo conventional autoantibody testing, including ANA, ASMA, and LKM-1. When conventional autoantibodies are positive, AIH is supported, and liver biopsy is recommended to confirm compatible or typical histological findings. In patients with negative or equivocal conventional autoantibodies, extended serological testing for SLA, LC1, and AMA may help support AIH or identify PBC/AIH overlap. AMA positivity should prompt evaluation for PBC or overlap syndrome, particularly when bile duct injury or loss is observed histologically. In patients with all-negative, atypical, or seronegative/equivocal findings, anti-DOK2 and anti-MRPS27 antibodies may provide additional serological support for AIH. Positivity for these emerging autoantibodies supports the diagnosis of AIH, whereas negativity does not exclude AIH; histological assessment and exclusion of alternative liver diseases remain essential.

To illustrate the potential clinical application of these emerging biomarkers, we propose a diagnostic algorithm that integrates conventional and novel autoantibodies (Figure 2). In patients with suspected AIH, conventional autoantibody testing, including ANA, ASMA, and anti-LKM-1 testing, is first performed after other causes of liver injury are excluded. If these conventional autoantibodies are negative, extended serological testing for antibodies such as anti-SLA and anti-LC-1 may be considered. In patients who remain seronegative but are clinically suspected of having AIH, evaluation of emerging autoantibodies, such as anti-DOK2 and anti-MRPS27 antibodies, may provide additional diagnostic clues. Histological confirmation by liver biopsy remains essential for the definitive diagnosis of AIH and for disease staging.

In this proposed workflow, testing for emerging autoantibodies is not intended to replace conventional diagnostic steps. Instead, their evaluation may be performed as second-line or adjunctive tests in patients who remain diagnostically ambiguous after standard serological evaluation. This approach may be particularly useful in patients who are negative for ANAs and ASMAs, as well as anti-LKM-1, anti-SLA/LP, and anti-LC-1 antibodies, but with persistent clinical suspicion for AIH. In such cases, positivity for anti-DOK2 or anti-MRPS27 antibodies could provide additional serological support and may prompt careful histological reassessment or closer therapeutic consideration. Conversely, negative results do not exclude AIH when clinical and histological findings are strongly suggestive. Thus, these biomarkers should be incorporated into a probabilistic diagnostic framework rather than used as binary diagnostic determinants.

## 8. Challenges and Barriers to the Clinical Implementation of Emerging Autoantibodies

### 8.1. Assay Standardization

Despite their promising diagnostic potential, several issues must be resolved before emerging autoantibodies can be adopted in routine practice. Assay standardization remains a major challenge, as differences in antigen preparation, assay platforms, cut-off values, and reference populations may affect diagnostic performance across studies. Assay standardization is the first prerequisite for clinical implementation. Diagnostic performance may vary depending on the antigen preparation method, protein conformation, assay platform, serum dilution, cut-off determination, and composition of control populations. For newly identified biomarkers, such as anti-DOK2 and anti-MRPS27 antibodies, differences between recombinant proteins, peptide fragments, and cell-free expressed proteins may influence antibody binding. In addition, cut-off values determined from healthy controls may not necessarily provide optimal discrimination in real-world clinical settings in which disease controls are more relevant. Therefore, standardized protocols and interlaboratory reproducibility studies are needed before these assays can be widely applied in routine practice.

### 8.2. External Validation

In addition, external validation in large and diverse cohorts is still limited, which may restrict the generalizability of the current findings. External validation is another essential step. Most emerging autoantibodies have been evaluated in limited cohorts, while diagnostic accuracy may differ depending on ethnicity, age, disease phenotype, treatment status, and the spectrum of disease controls. Most currently available studies on emerging AIH autoantibodies, including anti-DOK2 and anti-MRPS27 antibodies, were performed using retrospective cohorts. Control groups were always age- or sex-matched, and cut-off values were often derived internally. Future prospective, multicentre, externally validated studies using prespecified cut-off values are required. Validation studies should include patients with acute-onset AIH, AIH with normal serum IgG levels, seronegative AIH, overlap syndromes, drug-induced liver injury, viral hepatitis, MASLD, PBC, and PSC. Such studies are necessary to define the real-world sensitivity, specificity, PPVs, and NPVs of these biomarkers. In addition, validation in geographically and ethnically diverse cohorts is important for determining whether findings from Japanese or European cohorts are generalizable to broader AIH populations.

### 8.3. Clinical Positioning

The precise place of these biomarkers in clinical practice also remains uncertain. Although these markers may improve diagnostic sensitivity, particularly in patients with seronegative AIH or AIH with normal serum IgG levels, their integration into existing diagnostic algorithms has not yet been established. The clinical positioning of emerging autoantibodies also requires careful consideration. It remains unclear whether these biomarkers should be measured in all patients with suspected AIH or selectively used in patients who are negative for conventional autoantibodies. Their incorporation into diagnostic scoring systems would require robust evidence showing incremental diagnostic value beyond existing clinical, biochemical, immunological, and histological parameters. Until such evidence becomes available, these antibodies should be considered complementary tools rather than standalone diagnostic markers. Their greatest clinical value may lie in improving diagnostic confidence in patients with atypical or seronegative presentations.

### 8.4. Biological Interpretation

Finally, the biological significance of emerging autoantibodies remains incompletely understood. Some autoantibodies may participate directly in disease pathogenesis, whereas others may represent secondary immune responses following hepatocellular injury, apoptosis, mitochondrial stress, or antigen release. Distinguishing pathogenic antibodies from epiphenomenal markers is essential for understanding their clinical meaning. Longitudinal studies assessing changes in antibody titres before and after immunosuppressive therapy may clarify whether these markers reflect disease activity, the treatment response, or the risk of relapse. Integration with tissue-based analyses, including immunohistochemistry, imaging mass cytometry, and single-cell transcriptomics, may further define the relationship between circulating autoantibodies and intrahepatic immune microenvironments.

## 9. Molecular and Spatial Pathology of DOK2 and MRPS27 in Patients with AIH

Recent advances in imaging mass cytometry have enabled high-dimensional mapping of the immune cell architecture and cellular interactions in human liver tissue, providing unprecedented insights into the immune niches that underlie chronic inflammatory liver diseases, including AIH [28]. Immunohistochemistry and imaging mass cytometry revealed that DOK2 was expressed predominantly in CD68-positive macrophages and subsets of CD3-positive T cells within hepatic lobules and portal tracts [26]. Given the role of DOK2 as a negative regulator of receptor tyrosine kinase and Toll-like receptor signalling, its upregulation may represent a feedback mechanism to restrain excessive inflammation. Spatially resolved analyses thus link serological findings with the intrahepatic immune architecture.

Immunohistochemical analysis revealed weak MRPS27 expression in normal liver tissue, whereas AIH liver tissues exhibited strong granular cytoplasmic staining in injured hepatocytes with interface hepatitis and bridging necrosis. The MRPS27 signal was localized mainly to the cytoplasm and perinuclear area, which is consistent with mitochondrial localization, and was absent from the nuclei, cell membranes, inflammatory cells, and the biliary epithelium. Quantitative digital image analysis demonstrated that the H score was significantly greater in AIH tissues than in normal liver tissues. These findings suggest that MRPS27 expression is increased in injured hepatocytes in patients with AIH, although circulating anti-MRPS27 antibody titres may not directly reflect the severity of individual histological inflammatory features [27].

These tissue-based findings provide a conceptual bridge between circulating autoantibodies and intrahepatic immune pathology. Serological biomarkers alone cannot be used to identify the cellular source of inflammation or the spatial relationships among hepatocytes, macrophages, T cells, and bile duct epithelial cells. However, tissue-based technologies provide detailed spatial information but are not suitable for repeated monitoring in routine practice. Therefore, the integration of serum autoantibody profiles with spatial pathology may offer a complementary strategy for understanding disease heterogeneity in patients with AIH. In the future, combined serological and tissue-based approaches may help define biologically distinct AIH subgroups and may contribute to the development of more individualized diagnostic and therapeutic strategies.

## 10. Future Perspectives

Future research on serological biomarkers for AIH should focus on three key areas. First, multicentre studies are needed to validate emerging autoantibodies in clinically relevant populations, including patients with acute-onset AIH, AIH with normal serum IgG levels, seronegative AIH, and overlap syndromes. Second, the integration of serological biomarker profiles with histological and molecular data may improve the understanding of disease heterogeneity and pathogenesis. Third, future diagnostic strategies may benefit from multimodal approaches that combine conventional autoantibodies, emerging biomarkers, clinical features, and histological findings. In this context, anti-DOK2 and anti-MRPS27 antibodies may become useful components of a next-generation diagnostic panel for AIH.

Future studies should also examine longitudinal changes in the titres of emerging autoantibodies before and after immunosuppressive therapy. If antibody titres change in parallel with biochemical remission, relapse, or histological improvement, they may have potential utility not only as diagnostic markers but also as disease monitoring tools. In addition, the integration of serological biomarkers with single-cell transcriptomics, spatial proteomics, digital pathology, and AI-assisted histopathology may help identify distinct immunological subtypes of AIH [29,30,31]. Machine learning-based approaches may further enable the combined interpretation of conventional autoantibodies, emerging autoantibodies, serum IgG levels, biochemical profiles, clinical features, and histological findings [32]. Such integrated multimodal diagnostic models could clarify whether specific autoantibody profiles correspond to particular intrahepatic immune niches, treatment responses, or long-term outcomes. However, these models will require prospective validation, standardized data acquisition, and external multicentre testing before clinical implementation [30].

## 11. Conclusions

Recent advances in proteome-wide serological screening have expanded the landscape of candidate biomarkers for AIH. Among these, anti-DOK2 and anti-MRPS27 antibodies appear particularly promising as complementary markers for diagnostically challenging cases, including seronegative AIH and AIH with normal serum IgG levels. While liver biopsy remains indispensable for definitive diagnosis, testing for emerging autoantibodies may help refine the diagnostic workflow, increase diagnostic confidence, and provide novel insights into disease biology. Further validation, assay standardization, and integration with molecular pathology data will be essential to translate these biomarkers into routine clinical practice.

## Figures and Tables

**Table 1:** Comparative diagnostic characteristics of conventional and emerging autoantibodies in autoimmune hepatitis.

Autoantibody	Target Antigen	Reported Positivity/Diagnostic Tendency *	Specificity/Diagnostic Implication	Clinical Utility	Major Limitations
ANA	Nuclear antigens	Frequently positive in type 1 AIH Approximately 65% in meta-analysis	Limited specificity	First-line marker	False positivity in other liver or autoimmune diseases
ASMA	Actin/cytoskeletal antigens	Frequently positive in type 1 AIH 43–75% in type 1 AIH	Limited specificity	First-line marker	Assay variability; not disease-specific
anti-SLA/LP	tRNP(Ser)Sec	Low positivity Approximately 19% in meta-analysis	High specificity Approximately 99% in meta-analysis	Confirmatory/supportive marker	Low sensitivity
anti-LKM-1	CYP2D6	Mainly type 2 AIH rare in adult AIH	Relatively specific in appropriate context	Type 2 AIH marker	Rare in adults; geographic variability
anti-LC-1	FTCD	Mainly type 2 AIH 30% in type 2 AIH	Relatively specific in appropriate context	Type 2 AIH marker	Limited sensitivity
ASGPR	Asialoglycoprotein receptor	67–88% in type 1 AIH	Variable; reported positivity low in some controls	Supportive diagnosis; treatment responsiveness	Variable specificity; assay-dependent
PD-1	Programmed cell death-1	63% in type 1 AIH	AUC 0.80 vs. DILI	Adjunctive marker	Limited cohorts; requires external
PCK2	Phosphoenolpyruvate carboxykinase 2	~50% in type 1 AIH	91.5%	Adjunctive marker	Limited cohorts; requires external validation
PHGDH	D-3-phosphoglycerate dehydrogenase	80% (treatment-naïve); 61% (treated)	99%	Adjunctive marker	Limited cohorts; treatment status affects positivity
Nucleosome	DNA–histone complex	~71% in type 1 AIH	Not fully established in original AIH cohort; newer DILI comparison: 84.6%	Adjunctive marker	Limited specificity; reflects disease activity rather than AIH-specific diagnosis
RXFP1	Relaxin/insulin-like family peptide receptor 1	6–8% in type 1 AIH	No liver disease/healthy controls positive in validation subset; PhIP-seq hit criterion ≥ 99% specificity	Adjunctive marker	Supportive diagnosis; useful for seronegative AIH
HHV-6 U27/DIP2A cross-reactive antibodies	HHV-6 U27 gene product	6–8% in type 1 AIH	High specificity by PhIP-seq hit criteria	Adjunctive marker	Supportive diagnosis; useful for seronegative AIH
Polyreactive IgG	HIP1R/BSA-related reactivity	High positivity in seronegative/normal IgG AIH in reported cohorts 65–82% (type 1 AIH, treatment-naïve); 2–7% (type 2 AIH); 71% (normal IgG); 88% (ANA-negative)	Promising but requires validation	Adjunctive marker	Not antigen-specific; cohort-dependent
anti-DOK2	Docking protein 2	84% (type 1 AIH, treatment-naïve); 64–75% (normal IgG); 68–80% (ANA-negative)	Relatively high specificity in validation cohorts	Adjunctive marker for atypical/seronegative AIH	Limited cohorts; requires external validation
anti-MRPS27	Mitochondrial ribosomal protein S27	Moderate positivity in reported AIH cohorts ~61–62% (AIH); ~13% (non-AIH liver diseases/controls)	Moderate-to-high specificity in validation cohorts	Complementary adjunctive marker	Limited cohorts; requires external validation

* Reported positivity rates and diagnostic performance vary across studies because of differences in assay methods, cut-off values, patient populations, disease phenotypes, treatment status, and control group composition. Therefore, these values should not be interpreted as directly comparable sensitivity or specificity estimates across different autoantibodies.

**Table 2:** Diagnostic performance of anti-DOK2 and anti-MRPS27 antibodies and their combined use for autoimmune hepatitis.

Marker	Sensitivity (95% CI)	Specificity (95% CI)	PPV (95% CI)	NPV (95% CI)
Anti-DOK2 antibody	64.2% (52.2–74.6)	91.3% (84.2–95.3)	82.7% (70.3–90.6)	79.7% (71.5–85.9)
Anti-MRPS27 antibody	61.2% (49.2–72.0)	84.5% (76.3–90.2)	71.9% (59.2–81.9)	77.0% (68.4–83.8)
Anti-DOK2 or Anti-MRPS27 antibody	92.5% (83.7–96.8)	78.6% (69.8–85.5)	73.8% (63.5–82.0)	94.2% (87.1–97.5)

Abbreviations: CI, confidence interval; DOK2, anti-docking protein 2; MRPS27, mitochondrial ribosomal protein S27; NPV, negative predictive value; PPV, positive predictive value.

## Data Availability

The original contributions presented in this study are included in the article. Further inquiries can be directed to the corresponding author.
